# Application of QUAL2K Model to Assess Ecological Purification Technology for a Polluted River

**DOI:** 10.3390/ijerph120202215

**Published:** 2015-02-16

**Authors:** Wenting Zhu, Qian Niu, Ruibin Zhang, Rui Ye, Xin Qian, Yu Qian

**Affiliations:** State Key Laboratory of Pollution Control and Resource Reuse, School of the Environment, Nanjing University, Nanjing 210046, China; E-Mails: dtzwt1987@126.com(W.Z.); 0212.gloria@gmail.com(Q.N.); zhangrb88@126.com (R.Z.); rui.ye0220@163.com(R.Y.); yqian@nju.edu.cn(Y.Q.)

**Keywords:** ecological purification technology, degradation coefficient, hydraulic retention time, QUAL2K model

## Abstract

Industrialization and urbanization have caused water pollution and ecosystem degradation, especially in urban canals and rivers in China; accordingly, effective water quality improvement programs are needed. In this study, the Tianlai River in Jiangsu, China was taken as a research site, and a combination of ecological purification technologies consisting of biological rope, phytoremediation, and activated carbon were applied in a laboratory-scale study to examine degradation coefficients under dynamic water conditions. Coefficients were then input into the QUAL2K model to simulate various hypothetical scenarios and determine the minimum density of ecological purification combination and hydraulic retention time (HRT) to meet Grade V or IV of the China standard for surface water. The minimum densities for Grade V and IV were 1.6 times and 2 times the experimental density, while the minimum HRTs for Grade V and IV were 2.4 day and 3 day. The results of this study should provide a practical and efficient design method for ecological purification programs.

## 1. Introduction

In recent years, population growth associated with the rapid advancement of industrialization and urbanization has caused water pollution and ecosystem degradation, especially in urban canals and rivers in developing countries [1,2,3]. To solve these environmental problems, managers must select appropriate pollution load reduction programs for achieving particular goals, thus design of ecological purification programs can be a difficult task for water environmental managers.

There are a great many purification methods for polluted rivers, such as physical [4,5], chemical [6], and ecological [7,8]. Ecological purification technology is a promising approach to treatment of low-polluted water because it is inexpensive, has low maintenance requirements, and produces no harmful byproducts compared to the other methods [9,10]. Previous studies have shown the removal efficiency in stationary case [11] or in the single unit [12]. However, this is far from the circumstances of an actual river. Moreover, how to best determine the design parameters and predict the effects of purification is another key problem. In most literatures [13,14], estimates were made according to laboratory purification parameters, which have some obvious shortcomings such as uncertainty of removal effects by different ecological species and instability of the climate and surroundings of the actual effluent channel. As a result, quantitative methods are required to evaluate the effects of such purification projects. Currently, little attention has been given to the possibility of using water quality models to determine design parameters for engineering, to predict water quality [15,16]. The QUAL2K model is a flexible and accurate water quality model that has been widely applied in watershed pollutant control and water quality management. This model has been applied to medium-sized rivers with small width to depth ratios to track the fate and transport of targeted pollutants [17,18].

Owing to its large number of polluted tributaries, the water quality of the Yangtze River has decreased and it has become increasingly polluted. Accordingly, there is a great need for restoration of such small and medium rivers. As a result, the Tianlai River, in Nanjing, Jiangsu Province, is a low pollution river which was chosen as a research site. A combination of ecological purification technologies consisting of biological rope, phytoremediation, and activated carbon were applied in a laboratory-scale study to examine degradation coefficients under dynamic water conditions. Coefficients were then input into the QUAL2K model to simulate various hypothetical scenarios and determine the minimum density of ecological purification combination and hydraulic retention time (HRT) to meet Grade V or IV of the China standard for surface water. The main objectives of this study were to: (1) obtain the removal efficiency of each units and evaluate the effects of an ecological purify combination; (2) combine a water quality model with the designing of ecological purification programs; and (3) provide a methodological approach for the treatment of similar low polluted rivers.

## 2. Materials and Methods

### 2.1. Research Site

Tianlai River is 1290 m long, and its average depth and width are 2 m. The river is much longer in the longitudinal direction than the lateral and vertical direction, and pollutants in the river are evenly mixed in the water column. Tianlai River is a manually controlled river, which has a pumping station aside the river. The flow rate of the Tianlai River is controlled at 50 m^3^/h and the total river volume is approximately 24,912 m^3^. Concentrations of total nitrogen (TN), nitrate nitrogen (NO_3_^−^-N), ammonium nitrogen (NH_4_^+^-N), total phosphorous (TP), and orthophosphate (PO_4_^3−^-P) in the influent are 6.70 mg/L, 2.90 mg/L, 2.21 mg/L, 1.16 mg/L, and 0.42 mg/L, respectively. This river is on the campus of Nanjing University in China. There was little or no point pollution entering the river.

### 2.2. Experimental Design

**Figure 1:**
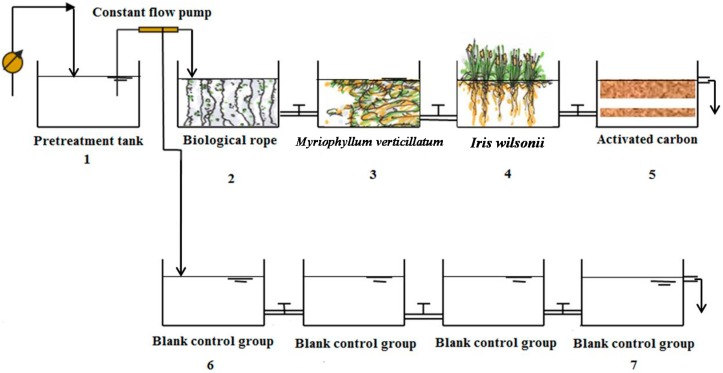
Schematic diagram of the experimental device.

The experimental site (32.1211° N, 118.9445° E) is located on the lower reaches of the Tianlai River at Nanjing University. Eight 96-L (40cm × 40cm × 60cm) colorless Perspex tanks were arranged in two parallel rows, one of which was used as an experimental group and another as the blank control (Figure 1). In the experimental group, the first tank contained 20 biological ropes that each had a length of 30 cm. The ropes were made of polypropylene and vinyl on with a specific surface area of 1.6 m^2^/m and a density of 1.24 kg/m^3^. The second tank contained 500 g *Myriophyllum verticillatum* L. telome derived from the Tianlai River. The third tank contained 20 *Iris wilsonii* plants. Plants were placed in an iron wire grid, and their roots were allowed to reach approximately 10 cm below the water surface. The last tank contained 2 kg of fresh activated carbon with a 1.5-mm-diameter column. Water from the Tianlai River was pumped into the pretreatment tank every hour using a timing device that was switched on for 3 min/h. The water in the pretreatment tank was then pumped through the two groups of water tanks at a flow rate of 60 mL/min using a constant flow pump. The hydraulic retention time (HRT) in this system was 4 day, which represents one day for each tank. The outdoor experiment was initiated on 16 April 2013 and completed on 14 May 2013. Overall, the outdoor experiment consisted of a 10-day stationary phase and a 20-day experimental period.

### 2.3. Sampling and Analysis

Water samples were collected from the pretreatment water tank (No. 1), experimental tanks (No. 2, 3, 4, 5), and the first (No. 6) and fourth tank (No. 7) of the control group. Three samples were collected from the upper, middle and lower part of the water column in each tank between 8:00 A.M. and 10:00 A.M., every day. The samples were then well-mixed and stored at 4 °C until analysis, which was conducted within 48 h. A portion of each sample was filtered through a Whatman GF/C 0.45 μm glass-fiber and analyzed for NH_4_^+^-N by the Nessler’s reagent colorimetric method, while TN and NO_3_^−^N were measured by ultraviolet (UV) spectrophotometry and nitrite nitrogen (NO_2_^−^-N) by the N-ethylenediamine colorimetric method. Unfiltered subsamples were analyzed for TN by alkaline potassium per-sulfate digestion-UV spectrophotometry, while PO_4_^3−^-P and TP were measured by ammonium molydate spectrophotometry [19]. Water temperature was recorded using a temperature light datalogger (UA-002-64, Hobo, Loveland, CO, U.S.), and turbidity was measured with a 2100Q portable turbidmeter (Hach, Loveland, Colorado, U.S.).

### 2.4. QUAL2K Model

The QUAL2K model is a one-dimensional, steady state water quality model that runs on Microsoft Windows. The model has been well-documented and is freely available from the United States Environmental Protection Agency (EPA) [20]. This model can simulate a number of water quality parameters, including temperature, pH, carbonaceous biochemical oxygen demand (CBOD represents the concentration of COD), sediment oxygen demand (SOD), dissolved oxygen (DO), organic nitrogen (ON), ammonium nitrogen (NH_4_^+^-N), nitrate nitrogen (NO_3_^−^-N), organic phosphorus (OP), inorganic phosphorus (IP), total nitrogen (TN), total phosphorus (TP), phytoplankton and bottom algae [21]. The model is applicable to well-mixed streams and considers dispersion and advection transport to occur only along the main flow direction (longitudinal direction). Advection-dispersion, dilution, interaction of chemicals and external import are considered in the model equation [22]. Application of the model extends to the presence of multiple pollution discharge and withdrawal locations and tributaries flowing into the main stream. In this context, degradation parameters of water quality indexes will vary with the density and HRT of ecological purification. Relationship curves between water quality, HRT and ecological material density were analyzed to determine the minimum density and HRT of the combination and ensure that the effluent reached Grade V or IV of the environmental quality standards for surface water.

Data input to the QUAL2K model include geometric data of the river system, hydraulic data, parameters, and data of the surroundings. First, the research river was divided into 12 sections, and their geometric and hydraulic data were input to the model. Then, headwater quality data such TN, NH_4_^+^-N, NO_3_^−^-N, and TP were input. Parameters of the “rates” sheet were changed to calibrate the model and construct a QUAL2K model for the river. After validating the constructed model, it could be used for water quality simulation. Through altering the degradation parameters of different water indices in each section, downstream water quality could be simulated.

## 3. Results and Discussion

### 3.1. Plant Growth and Biomass Production

In the biological rope tank, biofilm covered the rope with a thin layer at the beginning of the experiment, then turned dark green and became much thicker after 10 day. In the second tank, *M. verticillatum* sprouted and its weight increased by 32 g (6.4%). In the third tank, all plants grew steadily without obvious symptoms of toxicity or nutrient deficiency. Throughout the experiment, the 20 strains of *I. wilsonii* grew well in the tank, the average weight increased by 20 g (20%), and the leaf length increased by 2.5 cm (8.8%). Almost all *I. wilsonii* plants grew taller and stronger as their weight increased.

### 3.2. Removal Rate of nitrogen (N) and phosphorus (P)

**Figure 2:**
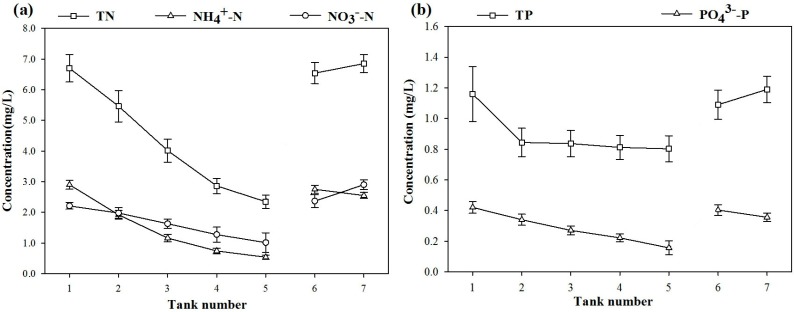
Average nitrogen (**a**) and phosphorus (**b**) indexes and their standard deviation along tanks.

Figure 2 shows the concentration in each tank, including the TN, NH_4_^+^-N, NO_3_^−^-N, TP and PO_4_^3−^-P. No significant difference (*p* < 0.05) was observed among the mean concentrations in each tank during the experiment.

The experimental group displayed effective removal of nutrients, but the concentrations of TN and TP in the last tank of the control group were higher than those in the first tank. Sims *et al.* observed the same phenomenon and suggested it might be due to accumulation of nutrients [23]. Because the water outlet was near the top (Figure 1), suspended particles remained in the bottom of the tank because of settling. Increased NO_3_-N was derived from the effect of hydrolysis and ammonification. The negative part of the control group was taken into consideration during calculation of the net removal rate. 

The net removal rate refers to the removal rate of the experimental group less that of a blank (Table 1). The results showed that the removal rate of NO_3_^−^-N was higher than that of other water quality indexes in every ecological unit, illustrating that the total ecological purification process had satisfactory NO_3_-N removal, reaching 47.8%. With the exception of the NO_3_^−^-N index, the biological rope unit showed the best TP removal, reaching 21.2%. *M. myriophyllum* as a submerged plant unit displayed the best NO_3_^−^-N removal, reaching 37.02%, while the *I. wilsonii* unit showed the best TN removal, reaching 30.3%, and the best NH_4_^+^-N removal, reaching 29.22%. The net removal rate of TP was low for the whole system, while the PO_4_^3−^-P removal rate was high, indicating that the net removal rate of OP in the system is quite low. However, the removal rate of OP in the control tank was high, indicating that OP particles were mainly separated from the water column via settling [24]. Conversely, dissolved PO_4_^3−^-P was removed through absorption by plants and microbial feeding [25]. The ecological purification technology showed stable N and P removal rates under dynamic conditions. The net removal rates of the whole system were 67.24%, 69.38%, 85.40%, 33.40%, and 47.55% for TN, NO_3_^−^-N, NH_4_^+^-N, TP and PO_4_^3−^-P, respectively.

**Table 1:** Net removal rate of each unit.

Indexes	Biological Rope Unit	*Myriophyllum verticillatum* Unit	*Iris wilsonii* Unit	Activated Carbon Unit
TN	16.07%	28.10%	30.33%	19.55%
NO_3_^−^-N	28.64%	37.02%	33.78%	25.03%
NH_4_^+^-N	17.68%	25.12%	29.22%	28.04%
TP	21.19%	3.96%	6.05%	4.17%
PO_4_^3−^-P	15.05%	16.51%	14.23%	25.52%

The results showed that the *I. wilsonii* and *M. verticillatum* units had excellent nitrogen removal. This was attributed to direct purification by *I. wilsonii* via absorption and enrichment, which directly removed pollutants, as well as indirect purification through the large surface area provided by the roots. The mechanisms of N and P removal by plants may include plant uptake, microbial uptake, and volatilization. Plant uptake has a direct contribution to nutrient content. This contribution to N and P has been reported in the range 25%–47% [26]. Moreover, the entire underwater surface of plants helps maintain an aerobic environment in the riverbed through oxygen transfer via roots and rhizome systems, and controls the growth of algae by restricting sunlight penetration [27].The biological rope unit showed excellent TP removal. The removal of phosphorus from this unit was primarily dependent on physical adsorption and deposition. Biological rope has a high filtering capacity for organic particles, while the microorganisms attached to the rope can facilitate hydrolysis and transformation. The results clearly demonstrate that vegetation and biological contact purification materials should be applied at the same time throughout the system, which supports previous findings [28,29,30]. They used integrations of plants and kinds of biological contact purification materials. The removal rates range from 19.5%–30.2% for TN, 22.2%–34.5% for TP. Some of the differences in removal efficacy between the current study and the others mentioned above may be attributable to differences in surface area coverage (5%–95%), differences in HRT, and/or study duration. Indeed, plants can contribute to treatment efficiency through plant uptake, the creation of an oxidized rhizosphere and adsorption-fixation reactions. Additionally, biological contact purification materials exert a removal effect by fixation of inorganic and organic particulates, sedimentation and deposition. The main reasons for N and P reduction in Tank 5 are as follows. Activated carbon has a large specific surface area compared with other fillers, such as burnt stone, vermiculite, and zeolite. The specific surface area of each gram of activated carbon reaches 1000 m^2^, which is why it has strong adsorption capacity. The pollutant interception process includes physical filtration, ion exchange, specific and non-specific adsorption, chelation, sedimentation reaction and others. Further, such material is important in providing additional media onto which microorganisms can attach.

### 3.3. QUAL2K Model Simulation

#### 3.3.1. Input Data and Model Parameters

**Figure 3:**
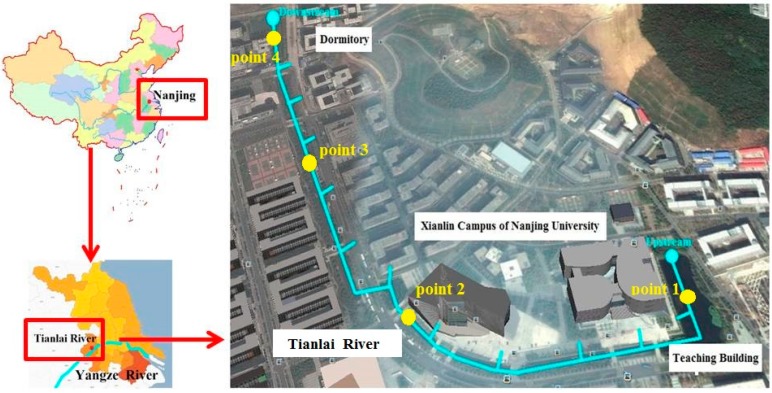
Location of the study area and sampling points in the Tianlai River.

The QUAL2K model can follow the specific circumstances of users to set the parameter values and transform the simulation equation to satisfy the user requirements. In this study, the input length of Tianlai River is 1290 m long. The river was divided into 12 water segments along the river flow direction (Figure 3), and no new calculation cells were divided in each segment. In the headwater of the river, average annual flow is 50 m^3^/h, average annual temperature is 18.7 °C, and pH is 7.2. Water quality from upstream and the environmental quality standards for surface water (GB3838-2002) for Grade IV and V are listed in Table 2.

**Table 2:** Comparison between upstream water quality of the Tianlai River and Grade V, IV of the China Standard for Surface Water (mg/L).

Classification	NO_3_^−^-N	NH_4_^+^-N	TN	TP	PO_4_^3−^-P	DO	SS
Concentration of upstream water quality	2.90	2.21	6.70	1.16	0.42	1.3	30
IV	--	1.5	1.5	0.3	--	3	--
V	--	2	2	0.4	--	2	--

Note: “--” indicates no value specified in the standard.

The extent of parameters (Table 3) that the QUAL2K model demanded were determined from a large number of studies including documentation for the stream water quality model QUAL2E, the QUAL2K user manual and the Environment Protection Agency guidance document[31].

**Table 3:** Parameters of the QUAL2K model for the Tianlai River.

Parameter	Value	Units	Symbol	Range
Carbon	40	g·C	g·C	30–50
Nitrogen	7.2	g·N	g·N	3–9
Phosphorus	1	g·P	g·P	0.4–2
Dry weight	100	g·D	g·D	100
Chlorophyll	1	g·A	g·A	0.4–2
ISS settling velocity	1	m/d	v_i_	0–2
O_2_ reaeration model	Internal	--	--	--
Slow CBOD:hydrolysis rate	0.3	/d	k_hc_	0–2
Fast CBOD:oxidation rate	0.4	/d	k_dc_	0.02–4.2
Organic N:hydrolysis	0.2	/d	k_hn_	0–5
Organic N:Settling velocity	0.05	m/d	v_on_	0–2
Ammonium:nitrification	0.8	/d	k_na_	0–10
Nitrate: denitrification	0.1	/d	k_dn_	0–2
Sed-denitrification transfer coefficient	0.05	m/d	v_di_	0–1
Organic P: hydrolysis	0.5	/d	k_hp_	0–5
Organic P: Settling velocity	0.6	m/d	v_op_	0–2
Inorganic P:settling velocity	0.27	m/d	v_ip_	0–2
Bottom algae:Maximum growth rate	10	mg·A/m^2^/d or /d	C_gb_	0–500
Bottom algae:First-order model carrying capacity	1000	mg·A/m^2^	a_b,max_	1000
Respiration rate	1	/d	k_rb_	0.05–0.5
Excretion rate	0.5	/d	k_eb_	0–0.5
Death rate	0.25	/d	k_db_	0–0.5
Light constant	50	langleys/d	K_Lb_	1–100
Ammonia preference	25	ug·N/L	k_hnxb_	1–100

The calculation time step was set to 5.6 min to ensure the model was maintained in the steady-state. The integration solution was handled with Euler’s method. The geometries and velocities of the river were used to determine the hydraulic characteristics. According to the relation formula between the mean velocity and flow rate, water depth and flow, the square law was used to determine coefficient for the velocity and flow rate, *a* is 2.45, *b* is 1.62, and to determine coefficient for water depth and flow coefficient, *c* is 0.79, *d* is 0.3. The longitudinal dispersion coefficient, D_L_, can also be obtained by the test simulation, tracer method or empirical formula. In this study, the river contains narrow ditches, so the Elder empirical formula was used to calculate D_L_. Based on this calculation, the D_L_ was 41.58.

Degradation parameters of water quality required for the model were changed according to different simulation scenarios. In this study, the degradation coefficient for ON, NH_4_^+^-N, NO_3_^−^-N, OP and IP were derived from the ecological purification experiment. Because the degradation process of water quality factors follows first-order reaction kinetics, the degradation parameters can thus be obtained. Degradation was calculated by [32,33]. This is the basic formula of pollution degradation in water quality model:

*C* = *C*_0_ × *e*^−k·*t*^ (1)

where *t* represents reaction time, *k* the degradation coefficient, *C* the concentration at time *t*, and *C*_0_ the initial concentration:

*RR* = (*C_0_* − *C*) × 100%/*C_0_* (2)

where *RR* represents removal rate. The degradation parameter itself would thus be represented by:

K = *t*^−1^ [ln (1 − *RR*)^−1^ × 100%] (3)

Parameter values are presented in Table 4. 

**Table 4:** Degradation parameters of the indexes in every unit.

Title Unit	Model Parameter (day^−1^)				
	NO_3_^−^-N	NH_4_^+^-N	ON	OP	IP
Biological rope	0.34	0.19	0.3	0.06	0.16
*Myriophyllum verticillatum*	0.46	0.29	0.3	0.04	0.18
*Iris wilsonii*	0.41	0.35	0.16	0.21	0.15
Activated carbon	0.29	0.33	0.28	0.04	0.29

#### 3.3.2. Model Calibration

**Figure 4:**
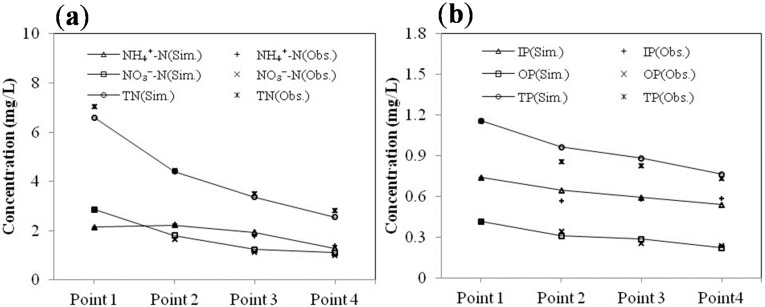
Water quality calibration results for the Tianlai River. (**a**). *N* indexes; (**b**). *P* indexes.

The QUAL2K model were calibrated by the observed data of the field experiment. Through trial and error, some input parameters in the model such as hydrolysis and sedimentation rates for the organic nitrogen and phosphorus, nitrification rate of NH_4_^+^-N and denitrification rate of NO_3_^−^-N were adjusted to be in a reasonable range and calibrated repeatedly until the simulation relative error for each parameter was within 10% (Figure 4 and Table 5). 

**Table 5:** Comparison of simulated and observed data (mg/L).

Sample outlet	NH_4_^+^-N		NO_3_^−^-N		TN	
	*Sim.*	*Obs.*	*Sim.*	*Obs.*	*Sim.*	*Obs.*
Point 1	2.150	2.150	2.854	2.854	6.594	7.033
Point 2	2.232	2.278	1.800	1.650	4.411	4.428
Point 3	1.926	1.790	1.232	1.121	3.368	3.490
Point 4	1.288	1.401	1.101	0.992	2.546	2.820
**Sample outlet**	**IP**		**OP**		**TP**	
	***Sim.***	***Obs.***	***Sim.***	***Obs.***	***Sim.***	***Obs.***
Point 1	0.742	0.742	0.419	0.419	1.161	1.161
Point 2	0.647	0.570	0.315	0.347	0.962	0.857
Point 3	0.593	0.580	0.289	0.259	0.882	0.829
Point4	0.542	0.589	0.224	0.244	0.766	0.733

Note: Sim. and Obs. represent simulated and observed data, respectively.

#### 3.3.3. Application of the Model

Comparison of the water quality of the Tianlai River and the environmental quality standards for surface water (GB3838-2002) indicated that the TN in the river was much higher than that required in the Grade V standard, the TP concentration was almost two times higher than the standard, and the NH_4_^+^-N concentration was slightly higher. Accordingly, the Tianlai River is a typical polluted river [2]. Therefore, effective purification technology in removing TN, TP and NH_4_^+^-N need to be investigated. There are two schemes which can improve the downstream water quality: A. by adding ecological purification combination; B. by prolonging HRT through utilization of natural water purification mechanism. The function of the QUAL2K model is to calculate the minimum density of ecological material or HRT required by the standard. 

##### ***A.*** ***Simulation of water quality after adding ecological purification combination at different density***

**Figure 5:**
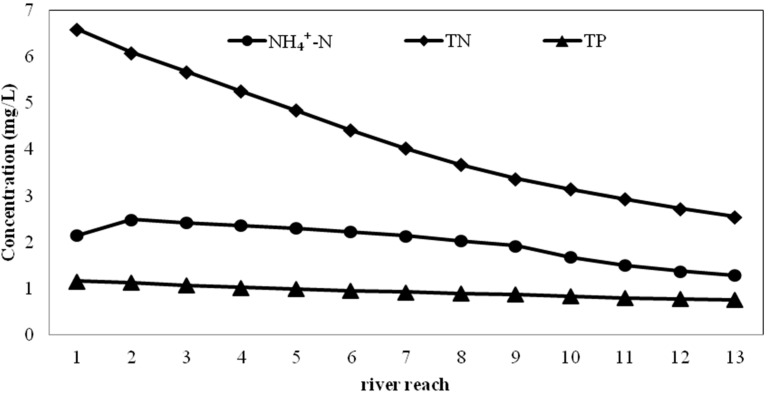
Simulation results of the water quality of Tianlai River under present conditions Simulation of water quality with ecological material at different densities.

The model simulated the water quality of the Tianlai River under the natural purification mechanism (Figure 5). As shown in Figure 5, the TN in the effluent decreased from 6.59 mg/L to 2.55 mg/L, while the NH_4_^+^-N decreased from 2.15 mg/L to 1.29 mg/L and the TP decreased from 1.16 mg/L to 0.77 mg/L. According to the surface water environmental quality standard (GB3838-2002), the NH_4_^+^-N, TN and TP concentrations of Grade V water are 2.0 mg/L, 2.0 mg/L and 0.4 mg/L, respectively. Therefore, effluent quality of the river cannot meet the standard if there is no further treatment.

**Figure 6:**
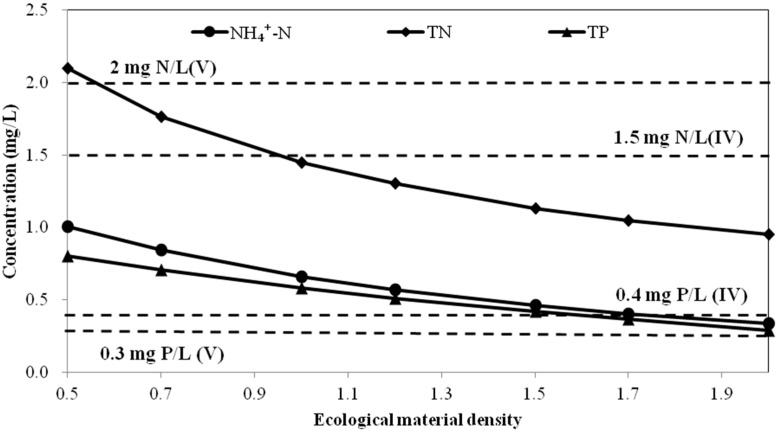
Relationship between effluent concentration and ecological material density. The dotted lines indicate Grade V and IV NH_4_^+^-N (2 mg/L, 1.5 mg/L), TN (2 mg/L, 1.5 mg/L) and TP (0.4 mg/L, 0.3 mg/L) levels.

Many experiments have shown that the nutrient removal rate was positively correlated with plant density and biomass in constructed wetlands [34,35]. A simulated experiment was conducted by White *et al.* [35] to compare the growth of four aquatic plants and their removal of total nitrogen and total phosphorus from eutrophication water under different plant densities. Wang *et al.* [34] investigated the purification efficiency of planting mixed species on eutrophic lake water with high, moderate and low input density. Both studies revealed that the removal rate improved with increasing density until the density became too high. As a result, we can assume that the removal rate is proportional to the ecological material density when the density is in a reasonable range. The density of each unit was set to 0.5, 0.7, 1.2, 1.5, 1.7 and 2.0 times the experimental cases, and the relationship curves between the effluent quality and ecological material density were simulated (Figure 6). In the experiment, 20 biological ropes that each had a length of 30 cm, 20 *I. wilsonii* plants, 500 g *M. verticillatum* and 2 kg of activated carbon were used to treat 96 L of polluted water, meaning that 62.5 m biological rope, 208 *I. wilsonii* plants, 5.3 kg *M. verticillatum* and 20.8 kg of activated carbon was needed in 1 m^3^ of polluted water. Two times that of the experiment means 125 m biological rope, 416 *I. wilsonii* plants, 10.6 kg *M. verticillatum* and 41.6 kg of activated carbon was needed in 1 m^3^ polluted water.

As shown in Figure 6, the effluent concentration decreased with increasing ecological material density. Ecological material density had the greatest influence on the TN concentration of the effluent, and less influence on the NH_4_^+^-N concentration, while the TP index was not sensitive. Therefore, to enable the effluent concentration to meet Grade V of the standard, the ecological material density must be 1.6 times that of the experimental conditions, while it must be two times the experimental conditions to generate effluent that meets Grade IV of the standard.

##### ***B.*** ***Simulation of water quality at different hydraulic retention time***

**Figure 7:**
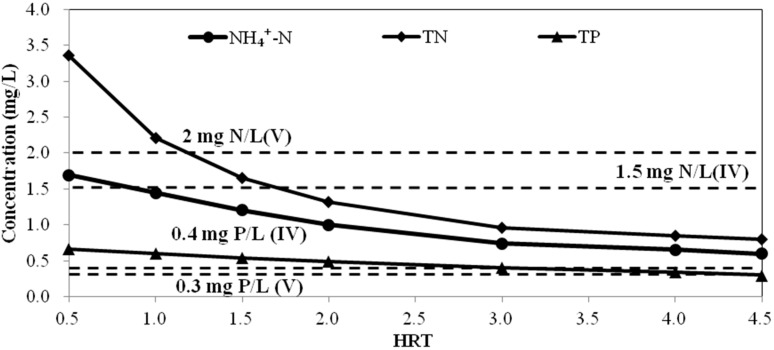
Relationship between effluent concentration and HRT. The dotted lines indicate Grade V and IV NH_4_^+^-N (2 mg/L, 1.5 mg/L), TN (2 mg/L, 1.5 mg/L) and TP (0.4 mg/L, 0.3 mg/L).

The flow rate of the Tianlai River is controlled at 50 m^3^/h and the total river volume is approximately 24,912 m^3^. Thus, the HRT is 20.85 day and the averaged HRT in each water segment is about 1.73 day. Accordingly, the HRT in the QUAL2K model was set to 0.5 day, 1 day, 1.5 day, 2 day, 2.5 day, and 3 day and the concentrations of TN, NH_4_^+^-N and TP were simulated. The results showed that the HRT had a great influence on water quality. The relationship curve between effluent water quality and HRT is presented in Figure 7.

Because TP is the limiting index, the total effluent can only meet the water quality standard if the TP agrees with the water quality requirements. The results indicated that the HRT should not be less than 2.4 day to enable the effluent concentration to reach Grade V of the standard, which corresponds to a flow rate of not more than 36.04 m^3^/h. To generate effluent that meets class IV of the standard, the HRT should be no less than 3 day, which corresponds to a flow rate of 28.83 m^3^/h.

## 4. Conclusions

In this study, the rationality of using the QUAL2K model to assess ecological purification technology applied to a polluted river (the Tianlai River) was investigated. A system designed for ecological purification was investigated and the unit degradation parameters were measured under dynamic conditions. The QUAL2K model for the Tianlai River was then established and validated. The conclusions can be summarized as follows: 

(1) The ecological purification technology showed stable N and P removal rates under dynamic conditions. Net removal rates of the entire system were 67.24%, 69.38%, 85.40%, 33.40%, and 47.54% for TN, NO_3_^−^-N, NH_4_^+^-N, TP and PO_4_^3−^-P, respectively.

(2)Changing the ecological material density of the combination in the QUAL2K model indicated that, to generate effluent that meets Grade V of the Chinese standard for surface water, the ecological material density should be 1.6 times that of the experiment, while it should be 2 times that of the experiment to meet Grade IV of the standard.

(3) Modifying the water retention time in the QUAL2K model revealed that the HRT should not be less than 2.4 day to enable the effluent concentration to reach Grade V of the standard, which corresponds to a flow rate of no more than 36.04 m^3^/h. To generate effluent that meets Grade IV of the standard, the HRT should be no less than 3 d, which corresponds to a flow rate of 28.83 m^3^/h.
